# Shift work and sleep duration are associated with adverse pregnancy outcomes in a predominantly Latinx population with high rates of obesity

**DOI:** 10.1371/journal.pone.0272218

**Published:** 2022-08-04

**Authors:** Jeannette M. Larson, Mihaela H. Bazalakova, Amy Godecker, Melanie DelBeccaro, Kjersti M. Aagaard, Kathleen M. Antony

**Affiliations:** 1 Department of Medical Education, University of Wisconsin-Madison School of Medicine and Public Health, Madison, WI, United States of America; 2 Department of Obstetrics and Gynecology, University of Wisconsin School of Medicine and Public Health, Madison, WI, United States of America; 3 Department of Neurology, Wisconsin Sleep, University of Wisconsin School of Medicine and Public Health, Madison, WI, United States of America; 4 Midwest Center for Women’s Healthcare, Park Ridge, IL, United States of America; 5 Division of Maternal-Fetal Medicine, Department of Obstetrics and Gynecology, Baylor College of Medicine, Houston, TX, United States of America; University of Lübeck: Universitat zu Lubeck, GERMANY

## Abstract

**Background:**

Shift work has been associated with adverse pregnancy outcomes. The objective of this study was to evaluate the association between sleep disturbances and adverse pregnancy outcomes.

**Methods and findings:**

This was a secondary analysis of a prospective study of participants enrolled in a prospective observational study wherein gravidae were screened for sleep apnea (2010–2012). A screening questionnaire with standard sleep apnea questionnaires as well as novel items about shift work and nocturnal sleep duration was administered at a prenatal care visit. Short sleep duration was defined as less than 7 hours. Prolonged sleep duration was defined as greater than 9 hours.

In a cohort of 1125 pregnant people, 9.4% reported shift work at the time of screening. Gravidae who reported shift work were more likely than gravidae who reported no shift work to develop preeclampsia (28.3% versus 13.0%, P<0.001), preeclamspsia with severe features (16.0% versus 8.5%, P = 0.010), gestational diabetes (28.3% versus 19.9%, P = 0.041), and a composite of adverse obstetric outcomes (61.3% versus 47.8%, P = 0.008). After adjusting for potentially confounding variables, shift work was associated with an increased risk for preeclampsia with (adjusted relative risk (aRR) 1.70, 95% CI 1.03–2.79, p = 0.036) and without (aRR 2.03, 95% CI 1.43–2.90, p<0.001) severe features, and gestational diabetes mellitus class A1 (aRR 1.47, 95% CI 1.05–2.05, p = 0.023) and class A2 (aRR 1.67, 95% CI 1.13–2.44, p = 0.009). Sleep duration was associated with gestational diabetes (31.3% among those with short sleep duration, 25.2% among those with normal sleep duration and 14.0% among those with prolonged sleep duration, P<0.001) and gestational diabetes class A2 (29.5%, 17.9%, and 10.1%, respectively, P<0.001). Gravidae with prolonged sleep duration experienced less composite adverse pregnancy outcomes at 42.6% compared to 57.4% for those with short sleep duration or 52.5% for those with normal sleep duration, P = 0.002.

**Conclusions:**

Shift work and sleep duration are both associated with adverse pregnancy outcomes. Further research on the impact of sleep disturbance on pregnancy outcomes is warranted.

## Introduction

According to the “restorative hypothesis,” sleep is an anabolic process of cellular and tissue regeneration [1]. Pregnancy is also considered an anabolic state, at least during the first trimester, with remarkable physiologic changes which collectively enable pregnancy adaptations, and these changes are likely to impact sleep in a bidirectional or reciprocal manner [2]. Studies of sleep characteristics during pregnancy suggest that total sleep duration increases or remains stable in the first trimester, followed by a decrease in sleep duration as pregnancy progresses [2,3]. Changes in sleep patterns during pregnancy are likely due to a combination of hormonal and physical factors [3]. Sleep disruptions during pregnancy may disrupt the restorative function of sleep, or may result from underlying pathology that impacts both sleep and physiology. Further external exposures, such as shift work, can also impact expected evolution of sleep homeostasis, timing, and duration during pregnancy. Shift work has been associated with adverse pregnancy outcomes such as preterm delivery, preeclampsia, lower birthweight and infants of small for gestational age [4–6]. Sleep duration has also been observed to bear a significant association with adverse maternal-fetal outcomes including late (near term) stillbirth and gestational diabetes mellitus (GDM) in women with prolonged sleep duration and GDM, pre-eclampsia, cesarean delivery, and preterm birth in women with short sleep duration [7–9]. Data on sleep deprivation and pregnancy outcomes are limited, though there have been mechanisms proposed to explain an observed association between sleep deprivation and preterm delivery [10].

We sought to examine the relationship between sleep and circadian disturbances, including shift work, short and long sleep duration, and sleep deprivation, and pregnancy outcomes. Our hypothesis was that shift work, sleep disruption (short sleep and/or long sleep duration), and sleep deprivation would be associated with adverse pregnancy outcomes, notably hypertensive disorders of pregnancy and gestational diabetes.

## Methods

The data for this study were collected from the Harris County Hospital District (now Harris Health System) between May 2010 and September 2012. The original study was approved by the Institutional Review Board at Baylor College of Medicine (IRB H-19183) [11,12]. This secondary analysis was determined to be exempt from approval by the Minimal Risk Health Sciences Institutional Review Board at the University of Wisconsin–Madison. Data were de-identified prior to use in this study. In the parent study, gravidae presenting to two community clinics and one tertiary clinic were approached for enrollment. Written informed consent documents in English or Spanish were signed by all participants as part of participation in the parent study. Consenting participants were administered a questionnaire (in English or Spanish), which comprised the Epworth Sleepiness Scale (ESS), the Berlin Questionnaire (BQ), and investigator-compiled questions about shift work, typical bedtimes and wake times for week days and weekends, falling asleep while driving, and napping. A separate study was performed to evaluate the association between adverse perinatal outcomes and screening measures of obstructive sleep apnea [11]. Gravidae of all gestational ages were recruited. Inclusion criteria also required gravidae to be between the ages of 18 and 50. Exclusion criteria were fatal fetal anomalies, multi-fetal gestation, and participants with significant underlying pulmonary or cardiac comorbidities. This present study examined obstetrical outcomes, thus participants who ultimately terminated their pregnancy or delivered at a non-study site were not included for outcomes analysis.

Participants were asked whether they were shift workers; if so, they were asked what shift they work. For the purpose of this investigation, shift work was defined by participant report of morning (starting before 6AM), evening (ending after 7PM) or night (ending after 12AM) shift which align closely with the definitions used by the Sleep Foundation [13]. Bedtime and wake time were assessed via the questionnaire separately for weekdays and weekends. Sleep duration was calculated by participant report of bedtime and wake time. For this study, short sleep duration was defined as a sleep duration of less than 7 hours; normal sleep duration was defined as 7–9 hours; and prolonged sleep duration was defined as greater than 9 hours [14,15]. Sleep deprivation was defined as >3 hours difference between weekday and weekend sleep duration [16–18]. Heightened sleep drive was also assessed indirectly by questions about falling asleep while driving, napping, and nap frequency.

Following delivery, each participant’s medical chart was reviewed and outcomes were individually extracted (MD & KMA6). Maternal outcomes included gestational hypertension, preeclampsia with and without severe features, gestational diabetes mellitus A1 and A2, preterm birth, and a composite of adverse obstetric outcomes which included gestational hypertension, preeclampsia, gestational diabetes mellitus A1 and A2, preterm premature rupture of membranes, placental abruption, chorioamnionitis, polyhydramnios, oligohydramnios, postpartum hemorrhage, and preterm delivery. The diagnosis of hypertensive disorders of pregnancy and gestational diabetes were consistent with definitions from the American College of Obstetrics and Gynecology during the study period (2010–2012) [19,20]. Gestational diabetes mellitus A1 (GDMA1) was defined as controlled with diet and gestational diabetes mellitus A2 (GDMA2) was defined by requiring medication [20]. Miscarriage was defined as spontaneous abortion occurring at or before 19 6/7 weeks of gestation [21]. Preterm premature rupture of membranes was defined as rupture of membranes at or before 36 6/7 weeks of gestation. Placental abruption, chorioamnionitis, polyhydramnios, oligohydramnios, and preterm delivery were diagnosed according to contemporary definitions [19,20,22,23]. This study was designed and started in 2010, therefore no core outcome set (COS) from the *Core Outcomes in Women’s and Newborn Health (CROWN initiative)* was used when designing the trial [24].

Neonatal outcomes included infants born small for gestational age, large for gestational age, and admission to level 2 or 3 nursery at birth. Infant small for gestational age was defined as birth weight less than the 10^th^ percentile and large for gestational age was defined as weight at birth greater than or equal to the 95^th^ percentile for gestational age [25,26].

Covariates considered in these analyses were maternal age, gravidity, ethnicity, and body mass index (BMI). Prepregnancy BMI categories were defined according to the National Institute of Health and the World Health Organization: underweight <18.5 kg/m^2^, normal weight 19–24.9 kg/m^2^, overweight 25–29.9 kg/m^2^, and obese >30 kg/m^2^ [27,28]. BMI was calculated using height and weight data that were collected prepregnancy. When prepregnancy weight was unknown, the earliest available weight during pregnancy was used.

Descriptive findings of study sample characteristics and outcomes by responses to questionnaire items are reported. Chi-squared testing was performed. Generalized linear models’ adjusted relative risks (aRRs) with 95% confidence intervals (Cis) were obtained among BMI categories, age categories, gravidity categories, and ethnicity for associations between questionnaire items and adverse maternal and neonatal outcomes using a modified Poisson regression approach [29]. Stata (Stata, College Station, TX, USA) was used for analyses.

## Results

**Fig 1:**
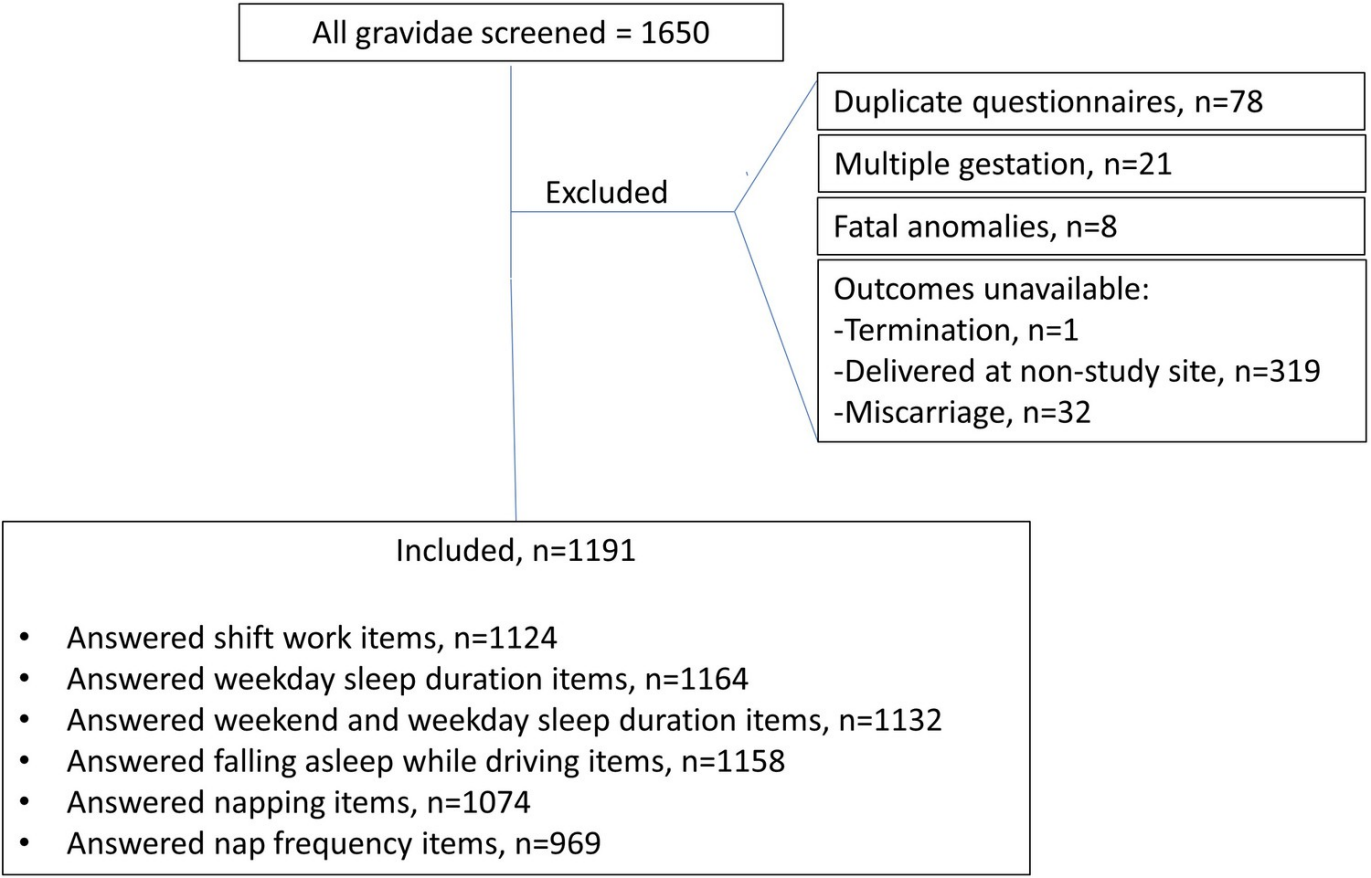
Gravidae included for analysis. Of 1650 completed questionnaires, outcomes were available for 1191 participants. The number included in each analysis is also shown.

1650 questionnaires were completed, and clinical information was analyzed for previously unidentified exclusion criteria. Duplicate questionnaires were excluded. Questionnaires with incomplete sleep duration, shift work, and sleep deprivation items were excluded from each respective analysis. (Fig 1)

As shown in Table 1, the majority of participants were Latina (90.4%). Over three-fourths of the participants were overweight (34.4%) or obese (37.1%). Shift work was associated with non-Latina ethnicity. Prolonged sleep duration (PSD) was associated with nulliparity, normal BMI, and maternal age less than 30. Overall, 12.1% (n = 135) reported shift work. 5.1% (n = 61) reported short sleep duration and 37.8% (n = 444) reported prolonged sleep duration. 17.4% (n = 199) of women were considered sleep deprived. 4.3% (n = 48) of women reported falling asleep while driving. 47.1% (n = 511) of women reported napping at the time of screening and 31.2% (n = 307) of women reported napping three or more times per week.

**Table 1:** a: Maternal characteristics of the study population by shift work (SW), sleep duration (short sleep duration (SSD), normal sleep duration (NSD), prolonged sleep duration (PSD)), sleep deprivation (SD), drowsy driving, and napping. b: Maternal characteristics of the study population by drowsy driving, and napping.

	SW +	SW–	p	SSD	NSD	PSD	P	SD +	SD –	p
	N = 106	N = 1019		N = 61	N = 659	N = 444		N = 199	N = 933	
**Maternal Age** <19 20–24 25–29 30–34 35–39 40+	3 (2.8%) 19 (17.9%) 27 (25.5%) 30 (28.3%) 21 (19.8%) 6 (5.7%)	80 (7.9%) 239 (23.5%) 282 (27.7%) 226 (22.2%) 150 (14.7%) 42 (4.1%)	0.132	2 (3.3%) 11 (18.0%) 13 (21.3%) 15 (24.6%) 16 (26.2%) 4 (6.6%)	29 (4.4%) 103 (15.6%) 182 (27.6%) 189 (28.7%) 122 (18.5%) 34 (5.2%)	57 (12.8%) 152 (34.2%) 122 (27.5%) 65 (14.6%) 37 (8.3%) 11 (2.5%)	**<0.001**	14 (7.0%) 35 (17.6%) 54 (27.1%) 41 (20.6%) 45 (22.6%) 10 (5.0%)	74 (7.9%) 226 (24.2%) 257 (27.6%) 215 (23.0%) 126 (13.5%) 35 (3.8%)	**0.020**
**Gravidity** 1 2 3–5 6+	19 (17.9%) 23 (21.7%) 53 (50.0%) 11 (10.4%)	185 (18.2%) 245 (24.0%) 512 (50.3%) 77 (7.6%)	0.752	7 (11.5%) 13 (21.3%) 33 (54.1%) 8 (13.1%)	87 (13.2%) 131 (19.9%) 385 (58.4%) 56 (8.5%)	125 (28.2%) 125 (28.2%) 166 (37.4%) 28 (6.3%)	**<0.001**	23 (11.6%) 45 (22.6%) 114 (57.3%) 17 (8.5%)	192 (20.6%) 220 (23.6%) 451 (48.3%) 70 (7.5%)	**0.019**
**Ethnicity** Latina Black Asian Caucasian Other	85 (80.2%) 17 (16.0%) 1 (0.9%) 3 (2.8%) 0 (0%)	937 (92.0%) 59 (5.8%) 14 (1.4%) 3 (0.3%) 6 (0.6%)	**<0.001**	55 (90.2%) 2 (3.3%) 1 (1.6%) 2 (3.3%) 1 (1.6%)	610 (92.6%) 35 (5.3%) 8 (1.2%) 2 (0.3%) 4 (0.6%)	400 (90.1%) 34 (7.7%) 6 (1.4%) 3 (0.7%) 1 (0.2%)	0.078	182 (91.5%) 13 (6.5%) 2 (1.0%) 2 (1.0%) 0 (0%)	858 (92.0%) 51 (5.5%) 13 (1.4%) 5 (0.5%) 6 (0.6%)	0.665
**BMI**^**1**^ <24.9 25.0–29.9 30+	28 (27.2%) 33 (32.0%) 42 (40.8%)	279 (28.7%) 334 (34.4%) 358 (36.9%)	0.737	19 (32.8%) 12 (20.7%) 27 (46.6%)	162 (25.6%) 227 (35.9%) 243 (38.5%)	145 (34.3%) 145 (34.3%) 133 (31.4%)	**0.004**	47 (24.7%) 59 (31.1%) 84 (44.2%)	270 (30.3%) 311 (34.9%) 311 (34.9%)	**0.049**

	Drowsy Driving +	Drowsy Driving –	p	Nap +	Nap –	P	Nap 3+	Nap <3	p
	N = 48	N = 1110		N = 511	N = 563		N = 307	N = 662	
**Maternal Age** <19 20–24 25–29 30–34 35–39 40+	5 (10.4%) 13 (27.1%) 11 (22.9%) 6 (12.5%) 11 (22.9%) 2 (4.2%)	84 (7.6%) 253 (22.8%) 304 (27.4%) 257 (23.2%) 166 (15.0%) 46 (4.1%)	0.369	57 (11.2%) 129 (25.2%) 114 (22.3%) 104 (20.4%) 81 (15.9%) 26 (5.1%)	30 (4 = 5.3%) 120 (21.3%) 171 (30.4%) 142 (25.2%) 75 (13.3%) 25 (4.4%)	**<0.001**	32 (10.4%) 83 (27.0%) 64 (20.9%) 64 (20.9%) 50 (16.3%) 14 (4.6%)	47 (7.1%) 147 (22.2%) 196 (29.6%) 159 (24.0%) 86 (13.0%) 27 (4.1%)	**0.019**
**Gravidity** 1 2 3–5 6+	11 (22.9%) 10 (20.8%) 22 (45.8%) 5 (10.4%)	207 (18.7%) 256 (23.1%) 564 (50.8%) 83 (7.5%)	0.731	122 (23.9%) 111 (21.7%) 232 (45.4%) 46 (9.0%)	81 (14.4%) 139 (24.7%) 301 (53.5%) 42 (7.5%)	**<0.001**	78 (25.4%) 67 (21.8%) 130 (42.4%) 32 (10.4%)	109 (16.5%) 167 (25.2%) 338 (51.1%) 48 (7.3%)	**0.001**
**Ethnicity** Latina Black Asian Caucasian Other	43 (89.6%) 3 (6.3%) 0 (0%) 0 (0%) 2 (4.2%)	1013 (91.3%) 71 (6.4%) 15 (1.4%) 7 (0.6%) 4 (0.4%)	**0.008**	432 (84.5%) 60 (11.7%) 9 (1.7%) 6 (1.2%) 4 (0.8%)	539 (95.7%) 16 (2.8%) 5 (0.9%) 1 (0.2%) 2 (0.4%)	**<0.001**	254 (82.7%) 41 (13.4%) 6 (2.0%) 3 (1.0%) 3 (1.0%)	623 (94.1%) 26 (3.9%) 8 (1.2%) 3 (0.5%) 2 (0.3%)	**<0.001**
**BMI**^**1**^ <24.9 25.0–29.9 30+	15 (32.6%) 9 (19.6%) 22 (47.8%)	305 (28.8%) 369 (34.8%) 385 (36.4%)	0.091	145 (29.8%) 155 (31.9%) 186 (38.3%)	147 (27.2%) 194 (35.9%) 199 (36.95)	0.373	94 (32.4%) 85 (29.3%) 111 (38.3%)	174 (27.4%) 225 (35.5%) 235 (37.1%)	0.133

SW = Shift work, SSD = Short Sleep Duration, NSD = Normal Sleep Duration, PSD = Prolonged Sleep Duration, SD = Sleep Deprived.

^1^ 51 participants did not have prepregnancy BMI available for analysis.

Drowsy Driving = Falling asleep while driving.

^1^ 51 participants did not have prepregnancy BMI available for analysis.

Table 2A shows the distribution of adverse maternal outcomes by shift work status, sleep duration, and sleep deprivation and associated questionnaire items. Unadjusted analyses indicate that shift work was associated with: preeclampsia with (p = 0.010) and without severe features (p<0.001), GDMA1 (p = 0.041), GDMA2 (p = 0.024), and the composite of adverse obstetric outcomes (p = 0.008). Similarly, in unadjusted analyses short sleep duration was associated with increased incidence of GDMA1 (p<0.001) and GDMA2 (p<0.001), while prolonged sleep duration was associated with decreased composite adverse obstetric outcomes (p = 0.002). Unadjusted analyses indicate that sleep deprivation was associated with large for gestational age infants (p = 0.015) and level 2/3 nursery admission (p = 0.048). Falling asleep while driving was associated with increased incidence of gestational hypertension (p = 0.032) and preeclampsia with (p = 0.018) and without severe features (p = 0.011). Napping was associated with increased incidence of preeclampsia with severe features (p = 0.047), preterm delivery (p = 0.001), and level 2/3 nursery admission (p = 0.006). Napping 3 or more times per week was associated with gestational hypertension (p = 0.036), preterm delivery (p = 0.011), and large for gestational age infants (p = 0.040).

**Table 2:** a: Unadjusted analysis of pregnancy outcomes by shift work (SW), sleep duration, sleep deprivation (SD), drowsy driving, and napping. b: Adjusted relative risks (aRRs) of pregnancy outcomes by shift work (SW), sleep duration, sleep deprivation (SD), drowsy driving, and napping.

	SW +	SW–	P	SSD	NSD	PSD	P	SD +	SD–	P	Drowsy Driving +	Drowsy Driving–	P	Nap +	Nap–	P	Nap 3+	Nap 3+	p
	N = 106	N = 1019		N = 61	N = 659	N = 444		N = 199	N = 933		N = 48	N = 1110		N = 511	N = 563		N = 307	N = 662	
**gHTN**	13 (12.3%)	99 (9.7%)	0.408	7 (11.5%)	69 (10.5%)	39 (8.78%)	0.601	21 (10.5%)	89 (9.54%)	0.677	9 (18.8%)	104 (9.36%)	**0.032**	60 (11.7%)	47 (8.33%)	0.062	38 (12.3%)	54 (8.14%)	**0.036**
**PreE**	30 (28.3%)	132 (13.0%)	**<0.001**	7 (11.5%)	98 (14.8%)	63 (14.2%)	0.761	33 (16.5%)	129 (13.8%)	0.327	13 (27.1%)	154 (13.9%)	**0.011**	78 (15.3%)	79 (14.0%)	0.560	51 (16.6%)	94 (14.2%)	0.323
**SPreE**	17 (16.0%)	86 (8.5%)	**0.010**	6 (9.84%)	63 (9.55%)	38 (8.56%)	0.843	21 (10.5%)	82 (8.79%)	0.445	9 (18.8%)	97 (8.73%)	**0.018**	57 (11.2%)	43 (7.62%)	**0.047**	36 (11.7%)	55 (8.30%)	0.088
**GDM**	30 (28.3%)	202 (19.9%)	**0.041**	19 (31.1%)	166 (25.2%)	62 (14.0%)	**<0.001**	44 (22.0%)	194 (20.8%)	0.704	9 (18.8%)	235 (21.3%)	0.689	104 (20.4%)	120 (21.2%)	0.709	60 (19.5%)	134 (20.2%)	0.809
**GDM A2**	24 (22.6%)	146 (14.4%)	**0.024**	18 (29.5%)	118 (17.9%)	45 (10.1%)	**<0.001**	33 (16.5%)	143 (15.3%)	0.678	8 (16.7%)	170 (15.3%)	0.797	76 (14.9%)	88 (15.6%)	0.740	42 (13.7%)	98 (14.8%)	0.650
**COOB**	65 (61.3%)	486 (47.8%)	**0.008**	35 (57.4%)	346 (52.5%)	189 (42.6%)	**0.002**	103 (51.8%)	448 (48.0%)	0.338	30 (62.5%)	540 (48.6%)	0.060	262 (51.3%)	268 (47.6%)	0.230	159 (51.8%)	309 (46.7%)	0.138
**SGA**	9 (8.49%)	131 (12.9%)	0.194	7 (11.5%)	70 (10.6%)	65 (14.6%)	0.133	26 (13.1%)	110 (11.8%)	0.615	6 (12.5%)	137 (12.3%)	0.974	71 (13.9%)	62 (11.0%)	0.152	38 (18.4%)	75 (11.3%)	0.636
**LGA**	4 (3.81%)	39 (3.84%)	0.987	3 (4.92%)	24 (3.65%)	14 (3.17%)	0.763	13 (6.57%)	28 (3.01%)	**0.015**	3 (6.25%)	40 (3.62%)	0.346	22 (4.32%)	16 (2.85%)	0.194	17 (5.57%)	19 (2.88%)	**0.040**
**NICU**	20 (19.0%)	168 (16.6%)	0.515	16 (26.2%)	101 (15.3%)	70 (15.8%)	0.085	41 (20.7%)	140 (15.0%)	**0.048**	11 (22.9%)	182 (16.5%)	0.240	104 (20.4%)	79 (14.1%)	**0.006**	61 (20.0%)	99 (15.0%)	0.052
**PTD**	20 (19.0%)	128 (12.6%)	0.063	13 (21.3%)	78 (11.9%)	53 (12.0%)	0.094	30 (15.2%)	110 (11.8%)	0.194	9 (18.8%)	140 (12.6%)	0.217	83 (16.3%)	56 (9.96%)	**0.001**	50 (16.4%)	70 (10.6%)	**0.011**

	SW	P	SSD	P	PSD	P	SD	P	Drowsy Driving	P	Nap +	P	Nap 3+	P
**gHTN**	1.24 (0.71–2.20)	0.449	1.18 (0.56–2.49)	0.671	0.74 (0.49–1.11)	0.141	1.14 (0.73–1.79)	0.568	2.06 (1.12–3.78)	**0.020**	1.33 (0.91–1.94)	0.136	1.43 (0.96–2.11)	0.075
**PreE**	2.03 (1.43–2.90)	**<0.001**	0.81 (0.41–1.62)	0.555	0.93 (0.67–1.29)	0.670	1.16 (0.80–1.67)	0.433	1.89 (1.15–3.12)	**0.012**	0.98 (0.72–1.33)	0.906	1.05 (0.76–1.46)	0.760
**SPreE**	1.70 (1.03–2.79)	**0.036**	1.10 (0.51–2.36)	0.814	0.91 (0.59–1.43)	0.691	1.20 (0.75–1.93)	0.442	2.01 (1.03–3.89)	**0.040**	1.29 (0.86–1.94)	0.223	1.21 (0.79–1.86)	0.381
**GDM**	1.47 (1.05–2.05)	**0.023**	1.23 (0.86–1.78)	0.259	0.77 (0.58–1.01)	0.059	0.93 (0.70–1.23)	0.602	0.86 (0.48–1.53)	0.613	1.04 (0.83–1.30)	0.740	1.05 (0.80–1.37)	0.728
**GDM A2**	1.67 (1.13–2.44)	**0.009**	1.63 (1.11–2.39)	**0.012**	0.85 (0.60–1.19)	0.343	0.90 (0.64–1.25)	0.517	1.03 (0.56–1.90)	0.928	1.05 (0.80–1.39)	0.708	1.03 (0.75–1.42)	0.849
**COOB**	1.26 (1.07–1.49)	**0.006**	1.11 (0.89–1.38)	0.372	0.84 (0.74–0.96)	**0.013**	1.02 (0.88–1.19)	0.770	1.27 (1.01–1.59)	**0.043**	1.05 (0.93–1.19)	0.402	1.09 (0.95–1.24)	0.232
**SGA**	0.62 (0.32–1.19)	0.153	0.94 (0.42–2.11)	0.889	1.37 (0.97–1.93)	0.074	1.22 (0.81–1.84)	0.346	0.95 (0.42–2.14)	0.904	1.13 (0.82–1.57)	0.453	0.93 (0.63–1.37)	0.720
**LGA**	1.04 (0.37–2.92)	0.934	1.39 (0.46–4.25)	0.561	1.17 (0.58–2.33)	0.662	1.83 (0.94–3.53)	0.074	1.76 (0.57–5.48)	0.327	1.68 (0.90–3.13)	0.104	2.15 (1.15–4.02)	**0.016**
**NICU**	1.06 (0.69–1.64)	0.783	1.61 (1.02–2.52)	**0.039**	1.15 (0.85–1.56)	0.371	1.26 (0.92–1.74)	0.150	1.35 (0.77–2.39)	0.294	1.31 (1.00–1.73)	0.054	1.18 (0.88–1.59)	0.263
**PTD**	1.37 (0.88–2.14)	0.164	1.69 (0.99–2.88)	0.054	1.15 (0.79–1.66)	0.471	1.22 (0.83–1.79)	0.319	1.50 (0.73–2.70)	0.309	1.45 (1.04–2.01)	**0.027**	1.32 (0.93–1.87)	0.117

SW = Shift work, SSD = Short Sleep Duration, NSD = Normal Sleep Duration, PSD = Prolonged Sleep Duration, SD = Sleep Deprived, Driving = Falling asleep while driving.

gHTN = Gestational hypertension, PreE = Preeclampsia, SPreE = Preeclampsia with severe features, COOB = Adverse composite obstetric outcome, SGA = Small for gestational age, LGA = Large for gestational age, NICU = NICU admission, PTD = Preterm delivery.

Table 2B shows the aRRs of adverse maternal and neonatal outcomes by shift work, sleep duration, and sleep deprivation and associated questionnaire items. After adjusting for confounding variables, shift work was significantly associated with preeclampsia (p<0.001), preeclampsia with severe features (p = 0.036), GDMA1 (p = 0.023), GDMA2 (p = 0.009), and adverse composite obstetric outcome (p = 0.006). Similarly, after adjusting for confounders, short sleep duration remained significantly associated with GDMA2 (p = 0.012) and prolonged sleep duration remained significantly associated with a decreased risk of adverse composite obstetric outcome (p = 0.013). Short sleep duration was also significantly associated with NICU admission at birth (p = 0.039). Sleep deprivation had no significant associations, while falling asleep while driving was significantly associated with gestational hypertension (p = 0.020), preeclampsia with (p = 0.040) and without severe features (p = 0.012), and adverse composite obstetric outcomes (p = 0.043). Napping remained significantly associated with preterm delivery (p = 0.027). Napping 3 or more times per week remained significantly associated with large for gestational age infants (p = 0.016).

## Discussion

Using both adjusted and unadjusted analyses, we found that measures of sleep disturbance were associated with differing primary and secondary adverse pregnancy outcomes. These findings are generally consistent with the diverse findings regarding sleep disturbances in the general population, where sleep deprivation and shift work have been associated with metabolic dysregulations, including diabetes and obesity, and adverse cardiac outcomes, including coronary artery disease [30,31]. These outcomes were chosen because they significantly impact maternal and newborn health. While no core outcome sets from the *CROWN initiative* were used due to this study being designed prior to the creation and publication of these order sets, relevant outcome sets for the outcomes described do exist [24,32].

Notably, our novel findings contrast to recent studies evaluating shift work and pregnancy outcomes; we found a statistically significant association between shift work and several maternal disorders that remained after adjusting for confounding variables [33]. Davari and colleagues found no association between shift work and adverse pregnancy outcomes (except for preterm birth) after adjustment for occupation and number of children [33]. In our study, the aRR of preeclampsia in gravidae participating in shift work was 2.03; in similar animal models, such sleep related changes have been hypothesized to be due to circadian rhythm disruption which affects clock gene expression that may regulate placental angiogenic factors such as VEGF [34–36]. Another notable finding is the lack of association between shift work and preterm birth, which corroborates the findings of some recent studies and meta-analyses [5,37,38] but is in contrast to others [6,33].

Short sleep duration was only associated with GDMA2 and NICU admission after adjustment for cofounders, which could be due to the relatively low proportion of our population reporting nightly sleep duration less than 7 hours. We also found that prolonged sleep duration demonstrated a non-significant decreased aRR of GDMA2. This conflicts with recent research suggesting an association between prolonged sleep duration and gestational diabetes mellitus [8]. In this analysis we found that prolonged sleep duration was significantly associated with a decreased risk for our composite adverse obstetric outcome. This could be due to overestimation of sleep time by subjective data collection, or it may be that prolonged sleep is indeed protective. Furthermore, many participants completed the questionnaire early in pregnancy, when sleep duration is expected to be prolonged [10]. Sleep duration and associated outcomes could be better elucidated in future studies with objective data collection or by repeated self-reported measurements throughout pregnancy.

Measures of higher sleep drive, such as falling asleep while driving, were associated with hypertensive disorders of pregnancy and adverse composite outcomes after adjusting for confounding variables. The associations of hypertensive disorders of pregnancy and adverse composite outcomes with falling asleep while driving are concordant with recent meta-analyses on sleep disturbance and pregnancy outcomes, suggesting that this question may be a better predictor of hypersomnolence than differences between weekday and weekend sleep durations as we utilized to measure sleep deprivation [9].

All napping was significantly associated with preterm delivery and frequent napping, three or more times weekly, was associated with large for gestational age infants. The divergence in associations between measures of sleep deprivation versus excessive sleepiness, as defined by drowsy driving and napping, suggests that different mechanisms beyond sleep deprivation alone may explain hypersomnolence in pregnancy and contribute to adverse pregnancy outcomes.

### Strengths and limitations

Strengths of our study include the large sample of participants with known maternal and fetal outcomes which were manually entered into the database by obstetric physicians (KMA6 and MD), prospective design, and adjustment for confounders. The main limitation of this study was the absence of objective measures and the lack of repeated data on sleep disturbance. The study was conducted with a questionnaire which was completed early in pregnancy for many participants which may fail to detect sleep disturbances with later onset in pregnancy or sleep disturbances exacerbated by pregnancy. This may also explain the low percentage of participants who reported short sleep duration and the high percentage of participants who reported prolonged sleep duration. In future studies, repeated data gathering, such as sleep journaling, or objective measurements, such as actigraphy or even use of daily wearable technology which track sleep, could contribute to the strength of evidence. The majority of the study population identified as Latina, which served as both a strength and a limitation in this study. The parent study was conducted in Houston, Texas at Ben Taub Hospital, which has a notably high Latina population [39,40]. As a strength, this study contributes to a limited body of evidence on sleep in pregnancy within the Latina population. However, this may limit generalizability to populations with different demographic makeups. Latina ethnicity is also notably a risk factor for gestational diabetes and possibly preeclampsia [41,42]. Future research in larger populations with varied demographic characteristics could further evaluate the applicability in the general population.

While there are numerous studies that demonstrate that sleep disordered breathing, obstructive sleep apnea, and snoring are associated with hypertensive disorders of pregnancy, in recent systematic reviews on sleep duration and shift work in pregnancy, it has been noted that data on association of these other sleep patterns with hypertensive disorders of pregnancy is limited [5,43]. This study contributes to the available evidence on this topic. Additionally, very little evidence is available on sleep deprivation in pregnancy, which this study adds to. In the context of prior evidence, our study suggests an association between sleep disturbances and adverse pregnancy outcomes. Proposed mechanisms for adverse effects of sleep disturbance include circadian rhythm disruption leading to hormonal dysregulation and increased oxidative stress leading to epigenetic changes [34,44]. Additionally, free radicals and oxidative stress following hypoxia and reperfusion have been implicated in placental damage in preeclampsia [45]. In animal models, melatonin has been shown to decrease oxidative injury to the placenta [46]. Given that melatonin production is influenced by environmental stimuli including the presence or absence of light as well as circadian rhythm, sleep disturbance may play a role in preeclampsia pathogenesis by disrupting melatonin function [47]. Another proposed mechanism for the association between sleep disturbance and preeclampsia is inflammation. Sleep disturbance has been associated with higher levels of inflammatory markers including C-reactive protein (CRP) and interleukin-6 (IL-6) [48]. Normal placentation is characterized by a profile of Th2 T-cells and anti-inflammatory cytokines such as interleukin-10 [49]. In contrast, preeclampsia is characterized by a predominance of Th1 T-cells and pro-inflammatory cytokines such as tumor necrosis factor (TNF) and interferon-gamma, which likely contributes to abnormal placentation and subsequent endothelial dysfunction [49,50]. Because sleep disturbance results in a pro-inflammatory state, it is plausible that the systemic inflammation associated with sleep disturbance contributes to development of pre-eclampsia.

Further studies are needed to clarify the relationship between distinct sleep disturbances and adverse pregnancy outcomes, as well as to evaluate potential mechanisms and clinical applications. Clinical research is needed to evaluate the most accurate screening measures for sleep disturbance and to evaluate whether treatment modifies the effects of sleep disruption on maternal and fetal health.

Our study adds to the available evidence on shift work, sleep duration, and sleep deprivation with adjustment for confounding factors. This analysis suggests that there may be an association between shift work and hypertensive disorders of pregnancy and GDM, and also an association between drowsy driving and hypertensive disorders of pregnancy and a composite of adverse pregnancy outcomes. This study also demonstrates the need for improved objective measures and unobtrusive methods for longitudinal assessment of sleep metrics and sleep disturbance in pregnancy.

## Supporting information

S1 Data (XLS)

S1 File (DOCX)
