# Supplementary material for: Shift work and sleep duration are associated with adverse pregnancy outcomes in a predominantly Latinx population with high rates of obesity
Source: PLoS One. 2022 Aug 4;17(8):e0272218. doi: 10.1371/journal.pone.0272218 (PMC9352044; doi:10.1371/journal.pone.0272218)
Supplement: S1 File — (DOCX) [file pone.0272218.s002.docx]

| SID | Numerical study ID |
| --- | --- |
| Ethnicity | Choices are Hispanic, Caucasian, Black, Asian, Indian (or whatever they enter). If they left it blank I consulted EPIC; there is a self-reported ethnicity option. |
| Sleep deprivation | Yes if >3 hr difference between weekday and weekend sleep time  0 if no  1 if yes |
| BMI | Pre-pregnancy BMI |
| BMI Class | 1 = <24.9  2 = 25.0-29.9  3 = >30.0 |
| Age | At time of survey |
| Gravidity |  |
| Nshift | Zero if no  1=Morning (Starts before 6 AM)  2=Day  3=Evening (Ends after 7 PM)  4=NIGHT (Ends after midnight) |
| Wkdayduration | Reported weekday sleep duration in hours |
| Driving | Drowsy driving  0 = no  1 = slight chance  2 = moderate chance  3 = high chance |
| Nap | 0 = no  1 = yes |
| Perwk | Naps per week |
| Ghtn | Gestational hypertension  0 = no  1 = yes |
| Pree | Pre-eclampsia  0 = no  1 = yes |
| Spree | Severe pre-eclampsia  0 = no  1 = yes |
| Gdm | Gestational diabetes mellitus  0 = no  1 = yes |
| A2dm | A2 gestational diabetes mellitus  0 = no  1 = yes |
| Nursery level | 1-3 |
| SGA5 | Small for gestational age  0 = no  1 = yes |
| LGA95 | Large for gestational age  0 = no  1 = yes |
| COOB | Adverse composite obstetric outcomes  0 = no  1 = yes |
| Gadelivery | Gestational age in weeks at delivery |
